# Supplementary material for: MicroRNA-483-5p Inhibits Hepatocellular Carcinoma Cell Proliferation, Cell Steatosis, and Fibrosis by Targeting PPARα and TIMP2
Source: Cancers (Basel). 2023 Mar 10;15(6):1715. doi: 10.3390/cancers15061715 (PMC10046356; doi:10.3390/cancers15061715)
Supplement: Supplementary file 1 [file cancers-15-01715-s001.zip › cancers-2244772-supplementary.pdf]

Supplementary Table S1. Characteristics of the study cohort. African American (AA); European American (EA); Asians American (AAM)

		AAM(N=10)	AA(N=14)	EA(N=16)
**Age**	*Mean (SD)*	56.8 (11.4)	59.4 (12.8)	65.6 (11.9)
**Gender**	*Male %*	100	78.6	62.5
**BMI**	*Mean (SD)*	15.3(12.4)	31.3(10.9)	28.4(15.0)
**HCV Serology**	*HCV Ab+ %*	30	57.1	31.2
	HCV RNA+ %	20	21.4	25
**HBV Serology**	*anti HBC+ %*	20	7.1	0
	HBs Ag+ %	20	28.6	0
**Smoking**	*Yes %*	80	64.3	50
	No %	20	35.7	50
**Alcohol**	*Yes %*	50	42.9	43.8
	No %	50	57.1	56.3
**MELD**	*Mean (SD)*	8.9 (3.7)	11.2 (4.7)	10.8 (4.7)
	MELD <10 %	50	50	50
**AFP**	*Median (IQR)*	3.6 (0.925)	11.2 (147.3)	5.4 (14.025)
**AST**	*Median (IQR)*	93 (108)	132 (140)	127.5 (171.8)
**ALT**	*Median (IQR)*	93 (81)	141(135)	101(128.5)
**Child Pugh score**	*Mean (SD)*	6.1 (1.7)	6.2 (1.7)	6.4 (1.3)
	Median (IQR)	5 (1)	6 (1)	6 (1.25)
**Child Pugh Class**	*A %*	70	78.6	62.5
	B %	20	7.1	37.5
	C %	0	7.1	0
**HCC Stage**	*Stage I %*	50	42.9	37.5
	Stage II %	30	21.4	25

Supplementary Table S2: Set of primers used in the current study.

**Human primers**
ACC FP: 5'-GCAGGTCACACGTCTCTTTAT-3'<br>ACC RP: 5'-CCAGCCTGTCATCCTCAATATC-3'
SREBP1 FP: 5'-GAGCCATGGATTGCACTTTC-3'<br>SREBP1 RP: 5'-AGCATAGGGTGGGTCAAATAG-3'
L-FABP FP: 5'-GGAATGTGAGCTGGAGACAA-3'<br>L-FABP RP: 5'-AGTTCGGTCACAGACTTGATG
SCD1 FP: 5'-CTCTTTCTGCTCTGCCATCTT-3'<br>SCD1 RP: 5'-CCCGACTTCACCTCCTTAAATC-3'
FASN FP: 5'-CTAGGTTTGATGCCTCCTTCTT-3'<br>FASN RP: 5'-GATGGCTTCATAGGTGACTTCC-3'
PPARγ FP: 5'-CTCAAACGAGAGTCAGCCTTTA-3'<br>PPARγ RP: 5'-GTGGGAGTGGTCTTCCATTAC-3'
FGF23: FP: 5'-TAACCCATCCCTCAGCAAAC -3'<br>FGF23: RP: 5'-GAGAGGCACAAGGAAGAGAAATA -3'
TIPM2: FP: 5'-CCGAGACAAAGAGGAGAGAAAG -3'<br>TIMP2: RP: 5'-CCTCCTGATACGGGTGCCATAA -3'
TGFβ: FP: 5'- GTAAGAAGCACCAGCTTGATGT-3'<br>TGFβ: RP: 5'-GTTGGAGCTGTCATCGAAGTAG -3'
Cx3Cr1 FP: 5'- GCCCTTCTGGACTCACTATTT-3'<br>Cx3Cr1 RP: 5'-CTTTGGCTTTCTTGTGGTTCTT -3'
AST FP: 5'-CGAATTTTGAGACTGGGATTAGA -3'<br>AST RP: 5'-CACTCTGGGTTGAGATGATGAA -3'
p21 FP: 5'-CGCTCTACATCTTCTGCCTTAG -3'<br>p21 RP: 5'- CGGGATGAGGAGGCTTTAAATA-3'
CYTOKERATIN-7: FP: 5'-GCTGTCCGGTGGTTAATTTC-3'<br>CYTOKERATIN-7: RP: 5'-CAACAAGTTTCCTCCCTCATC-3'
MMP2 FP: 5'-CCGTGGTGAGATCTTCTTCTTC-3'<br>MMP2 RP: 5'-GGTTCTCCAGCTTCAGGTAATAG-3'
PPARa FP: 5'-GTTCTGGAAGCTTTGGCTTTAC-3'<br>PPARa RP: 5'-GCGTGGACTCCGTAATGATAG-3'
NOTCH1 FP: 5'-CGTCATCTCCGACTTCATCTAC-3'<br>NOTCH1 RP: 5'-CTCCCTGTTGTTCTCCATATCT-3'
NOTCH2 FP: 5'-ACCGACAGACAAATCAGGTAAG-3'<br>NOTCH2 RP: 5'CTTCTCCTTGGGCCAGTTAAT-3'
NOTCH3 FP: 5'-GGATGGCGTCAACACCTATAA-3'<br>NOTCH3 RP: 5'-GGCAGGCACAGTAGAAAGAA-3'
NOTCH4 FP: 5'- CTGCGATAATGCGAGGAAGATA-3'<br>NOTCH4 RP: 5'-CTCCTGTGGCCTGTCTTATTT-3'
HES1 FP: 5'- GCCAGTTTGCTTTCCTCATTC-3'<br>HES1 RP: 5'- AGTGCATGGTCAGTCACTTAATA-3'
GAPDH FP: 5'- CCACCCAGAAGACTGTGGAT -3'<br>GAPDH RP: 5'- GTTGAAGTCAGAGGAGACCACC -3'

**Mouse primers**
mTGFβ FP: 5’-CCAGATCCTGTCCAAACTAAGG-3’<br>mTGFβ RP: 5’-CTTCCCGAATGTCTGACGTATT-3’
mTIMP2 FP: 5’-GGTCACAGAGAAGAGCATCAA-3’<br>mTIMP2 RP: 5’-CTGCCTGTAGCAAGGATCAA-3’
mp21 FP: 5’-CAGACCAGCCTGACAGATTT-3’<br>mp21 RP: 5’-TAGGACTGTTCCTCCGGTATAG-3’
mMMP2 FP: 5’-GCTCTGTCCTCCTCTGTAGTTA-3’<br>mMMP2 RP: 5’-CCCTCCTAAGCCAGTCTCTATTA-3’
mAST FP: 5’-CCCAAGCAGGTCGAGTATTT-3’<br>mAST RP: 5’-CAGCTAGGTACAGCTAGGTTTG-3’
mNotch1 FP: 5’-CTCATTCTACCTGCACCCTAAC-3’<br>mNotch1RP: 5’-GCTGTCACTCTTCCTGCTAAA-3’
mNotch2 FP: 5’-GAATGGAGGAACGTGCAATAAC-3’<br>mNotch2RP: 5’-TTCATACACGGCTTGGAGATAC-3’
mNotch3 FP: 5’-GCCASTGTCCTCTTGGTTATAC-3’<br>mNotch3RP: 5’-GCTGACACTCATCCACATCTT-3’
mNotch 4 FP: 5’-GAGGTTTGCCAGCTCCTATT-3’<br>mNotch 4 RP: 5’-CTACTGTCCTGGGCATCTTTATC-3’
mGAPDH FP: 5’-GGAGAAACCTGCCAAGTATGA-3’<br>mGAPDH RP:-5’-CCTGTTGCTGTAGCCGTATT-3’

Figure S1

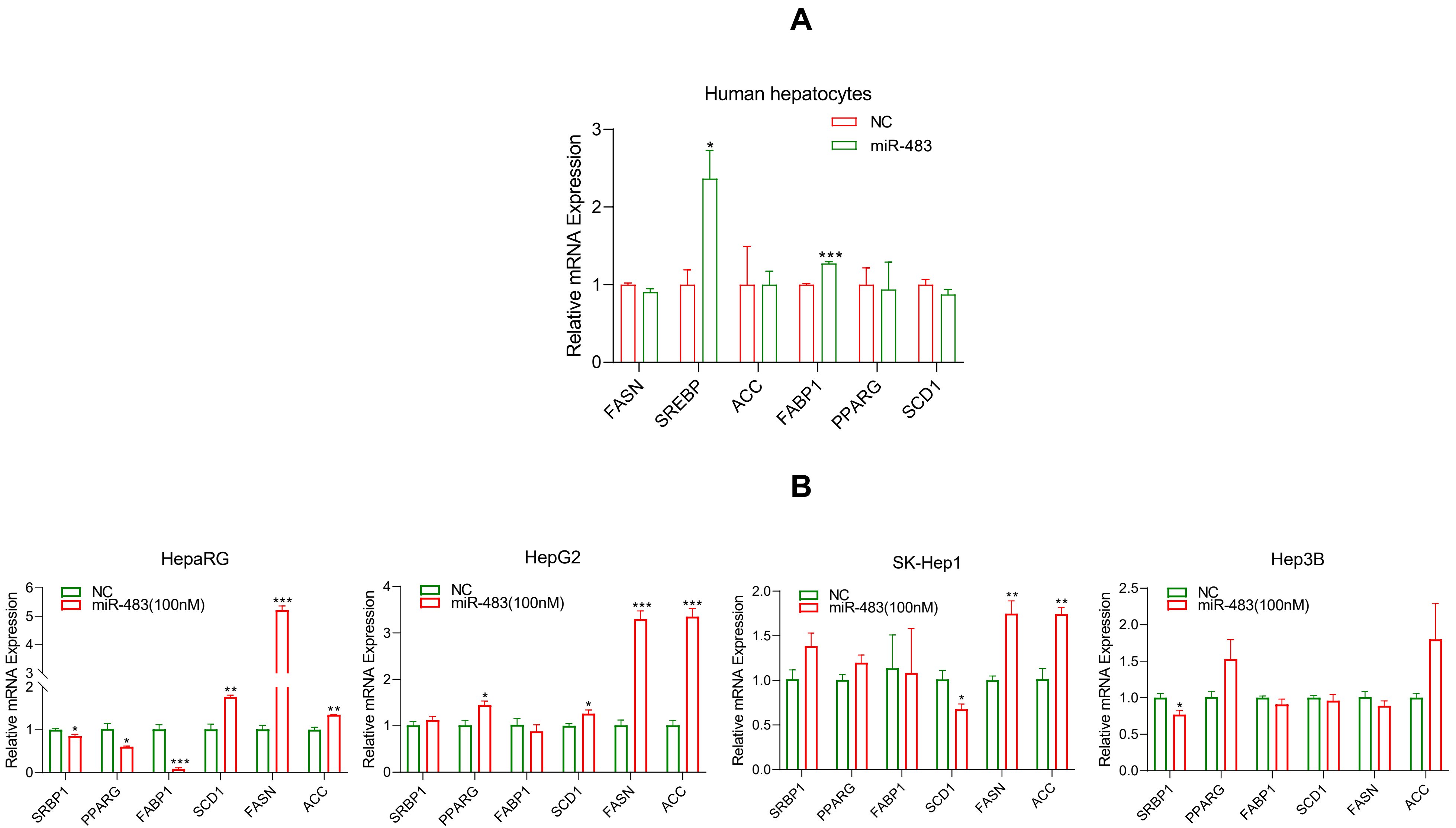

Figure S1: Effect of miR-483 on the expression of *FASN*, *SREBP1*, *ACC*, *FABP*, *PPARγ, and SCD1*. (**A**) Human primary hepatocytes were transfected with NC (100 nM) or miR-483 mimic (100 nM) as indicated for 30h and expression of *FASN*, *SREBP1*, *ACC*, *FABP*, *PPARγ,* and *SCD1* were analyzed by RT/qPCR. (**B**) HepaRG, HepG2, SK-Hep1, and Hep3B cells were transfected with NC (100 nM) or miR-483 mimic (100 nM) as indicated for 30h, and expression of *FASN*, *SREBP1*, *ACC*, *FABP*, *PPARγ,* and *SCD1* were analyzed by RT/qPCR. *$P < 0.05$, **$P < 0.01$, ***$P < 0.001$ compared with NC transfected cells.

Figure S2

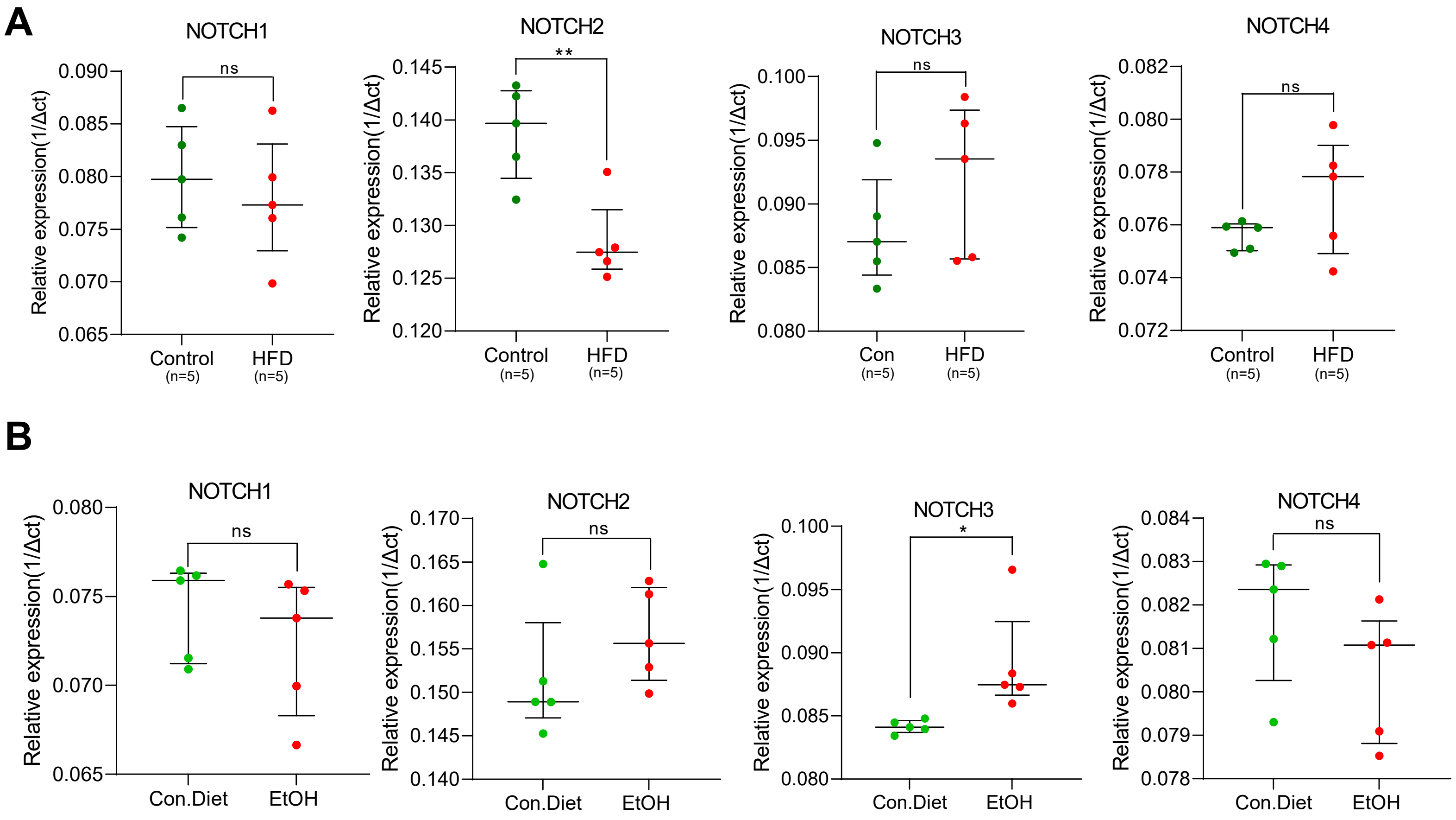

Figure S2: Expression of *Notch 1*, *Notch 2*, *Notch 3*, and *Notch 4* in the liver of NAFLD and AFLD mice model. (A&B) Male C57BL/6J mice pair-fed with control diet (n=5) or HFD for 16 weeks or EtOH diet (n=5) for 8 weeks, total RNAs from liver tissues were isolated and the expression of *Notch 1*, *Notch 2*, *Notch 3,* and *Notch 4* gene expression were analyzed by RT/qPCR. $*P < 0.05$, $**P < 0.01$, compared with control diet-fed mice. Ns- not significant.

Uncropped Western blots

Figure 1 D

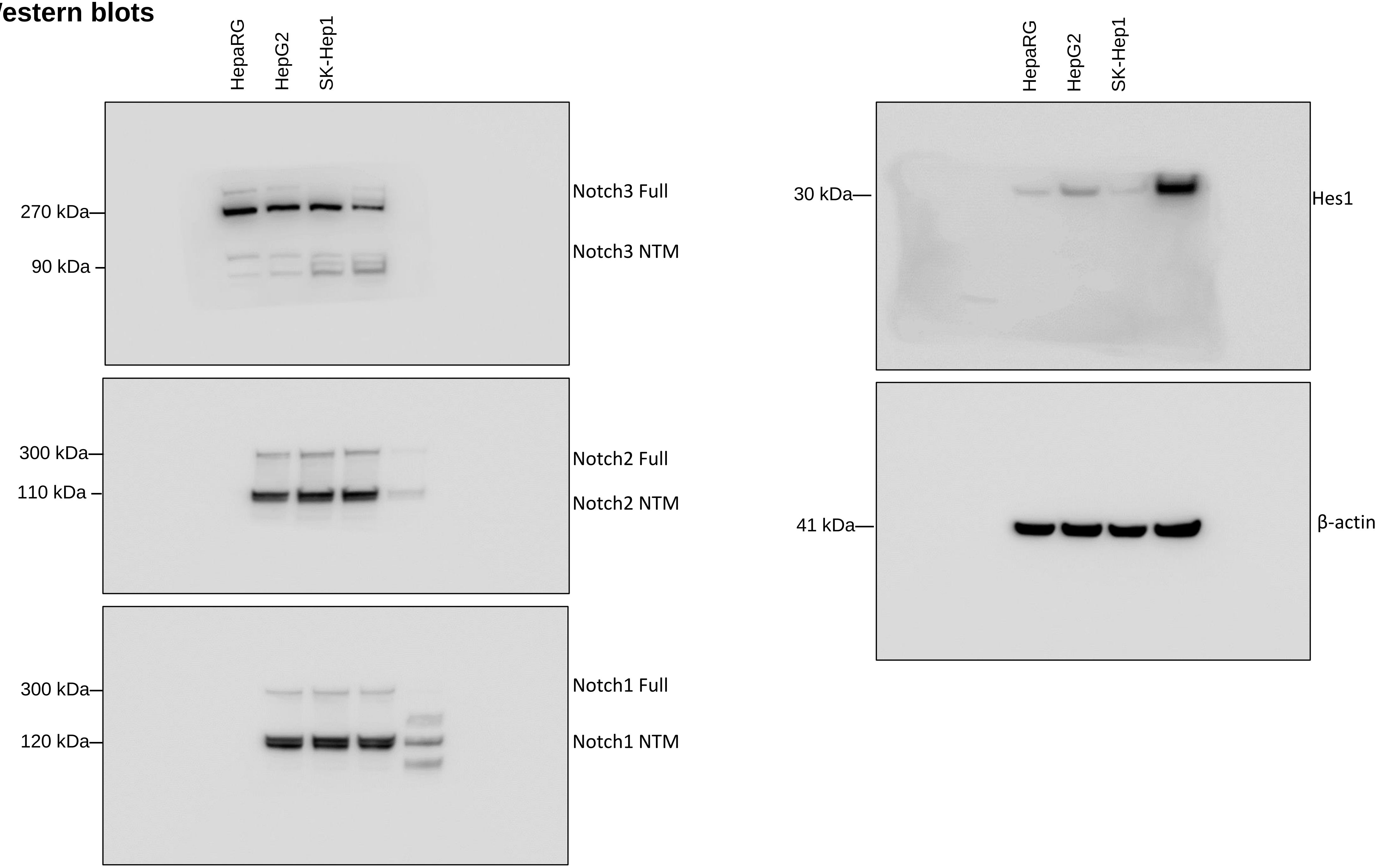

Figure 2 D

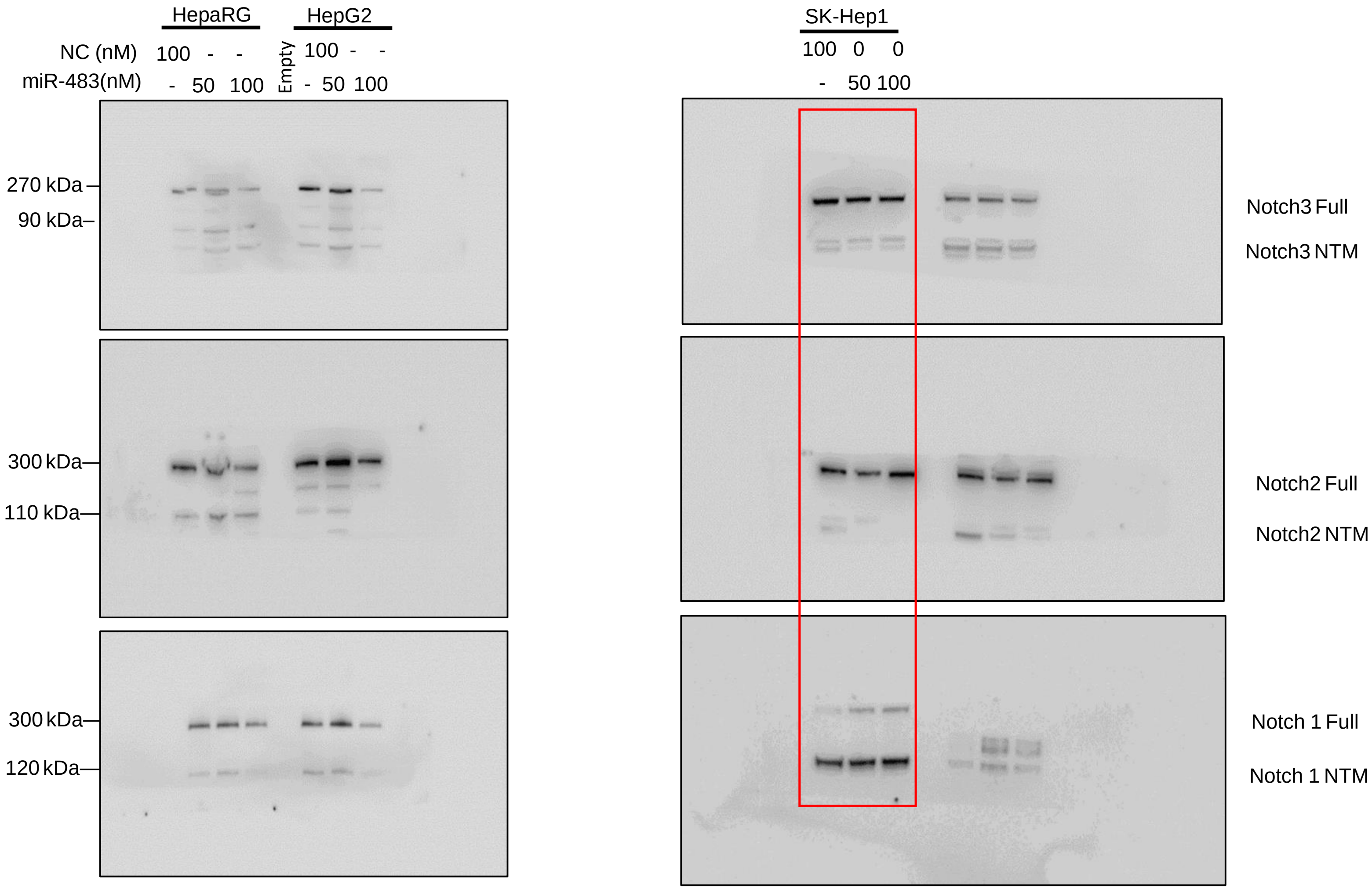

HepaRG
HepG2
NC (nM) 100 - -
miR-483(nM) - 50 100
Empty
100 - -
- 50 100
270 kDa
90 kDa
300 kDa
110 kDa
300 kDa
120 kDa
SK-Hep1
100 0 0
- 50 100
Notch3 Full
Notch3 NTM
Notch2 Full
Notch2 NTM
Notch 1 Full
Notch 1 NTM

Figure 2 D

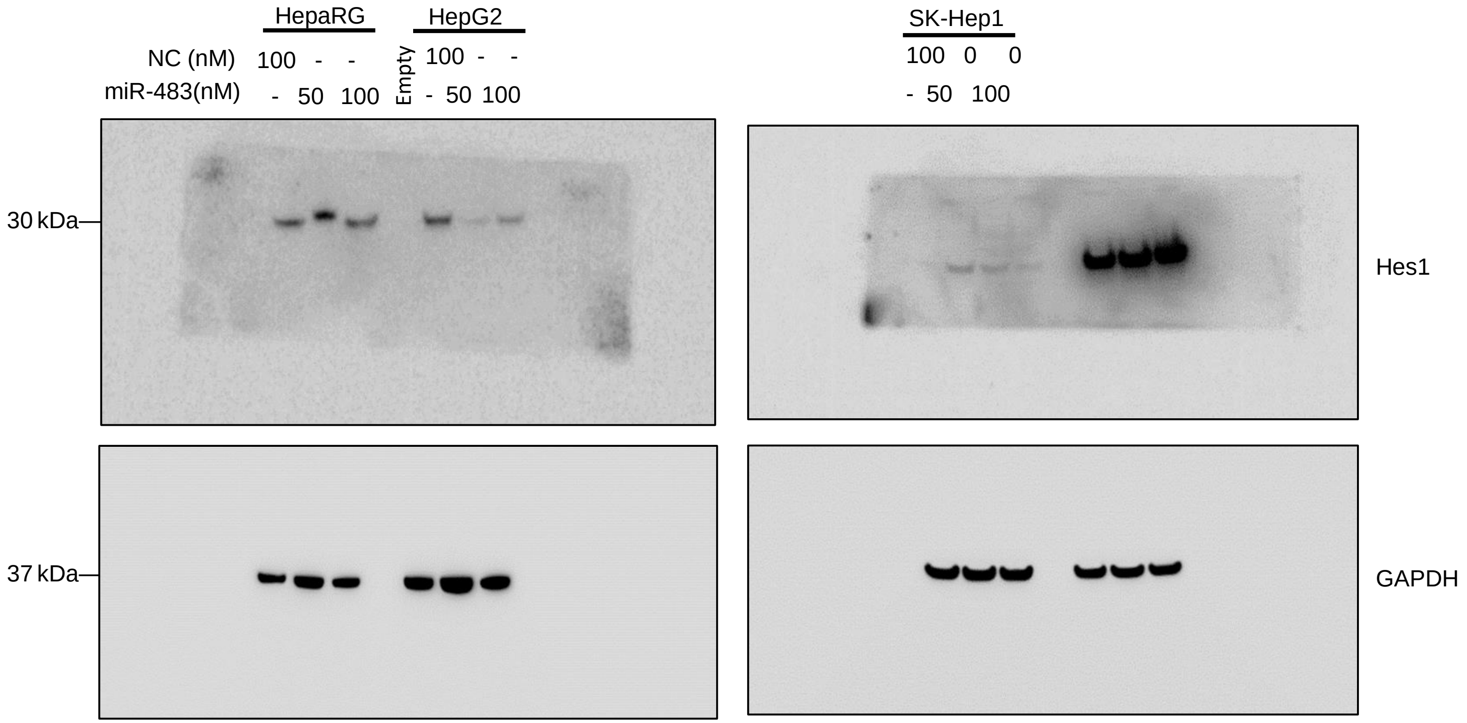
HepaRG
HepG2
SK-Hep1
NC (nM) 100 - -
miR-483(nM) - 50 100
Empty
100 - -
- 50 100
100 0 0
- 50 100
30 kDa
37 kDa
Hes1
GAPDH

Figure 2 E

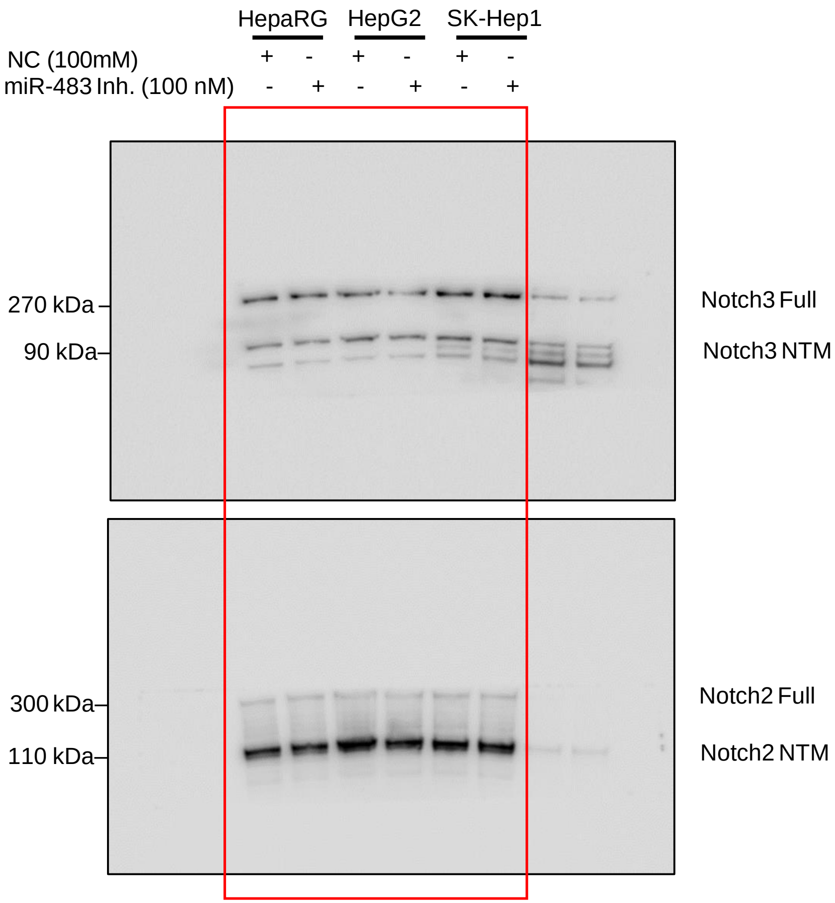
HepaRG
HepG2
SK-Hep1
NC (100mM)
+ - + - + -
miR-483 Inh. (100 nM)
- + - + - +
270 kDa
90 kDa
Notch3 Full
Notch3 NTM
300 kDa
110 kDa
Notch2 Full
Notch2 NTM

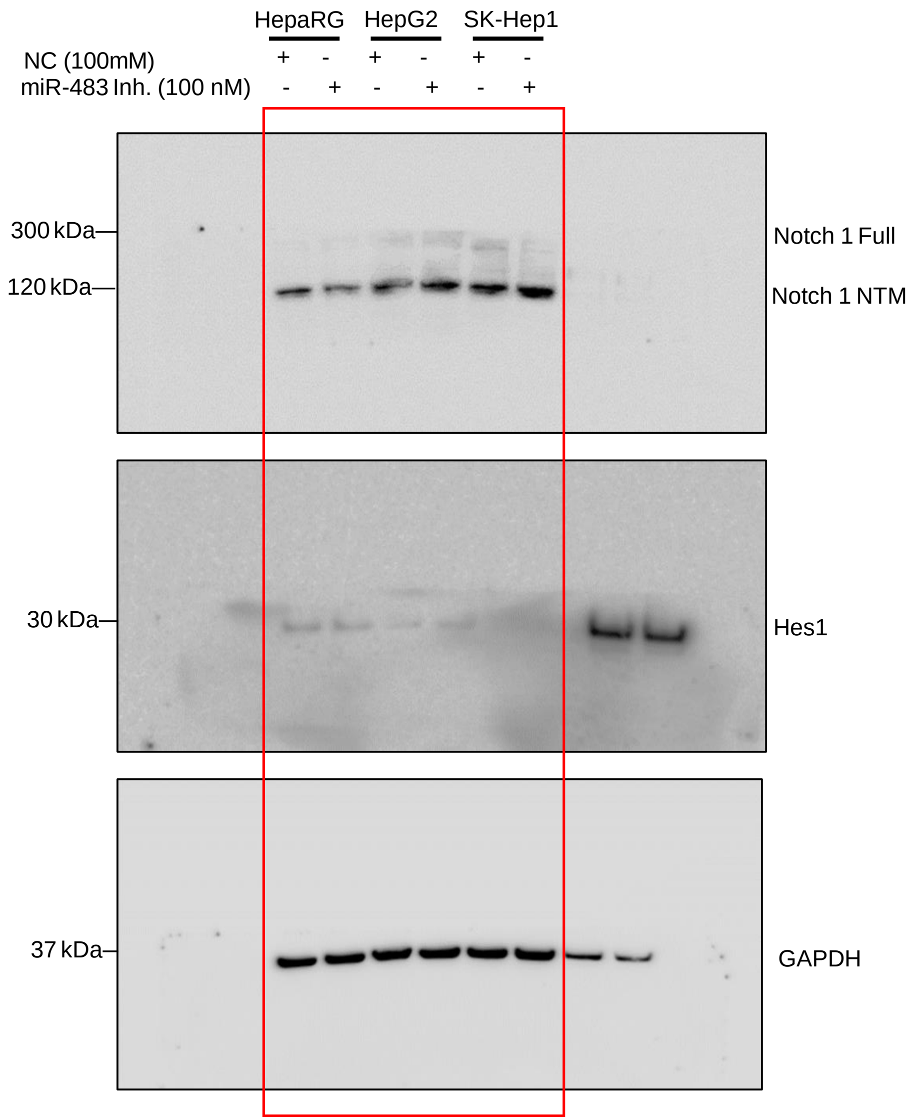
HepaRG
HepG2
SK-Hep1
NC (100mM)
+ - + - + -
miR-483 Inh. (100 nM)
- + - + - +
300 kDa
120 kDa
Notch 1 Full
Notch 1 NTM
30 kDa
Hes1
37 kDa
GAPDH

Figure 3 D

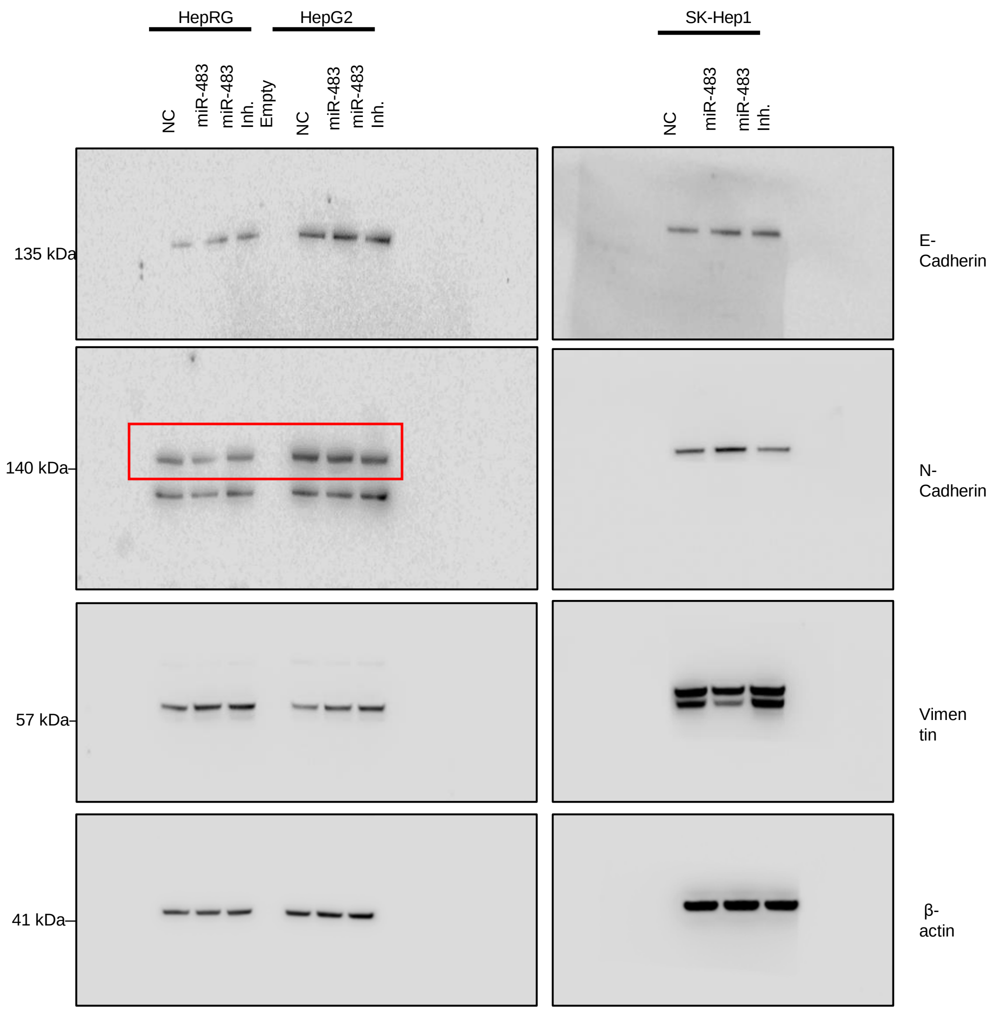
HepRG
HepG2
SK-Hep1
NC
miR-483
miR-483 Inh.
Empty
NC
miR-483
miR-483 Inh.
NC
miR-483
miR-483 Inh.
135 kDa
140 kDa
57 kDa
41 kDa
E-Cadherin
N-Cadherin
Vimentin
β-actin

Figure 3 E

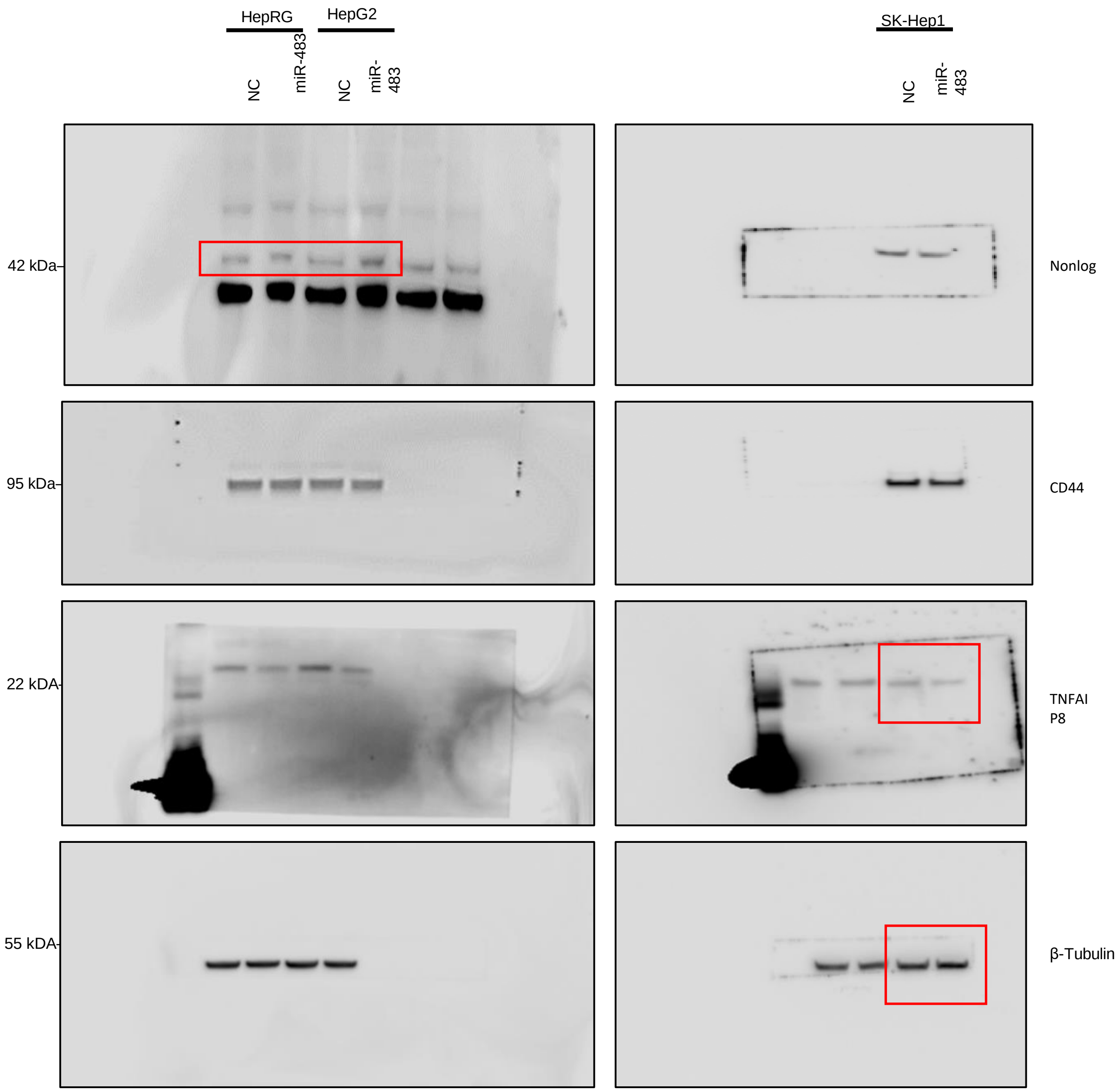

HepRG
HepG2
NC
miR-483
NC
miR-483
SK-Hep1
NC
miR-483
42 kDa
95 kDa
22 kDA
55 kDA
Nonlog
CD44
TNFAI P8
β-Tubulin

Figure 3 F

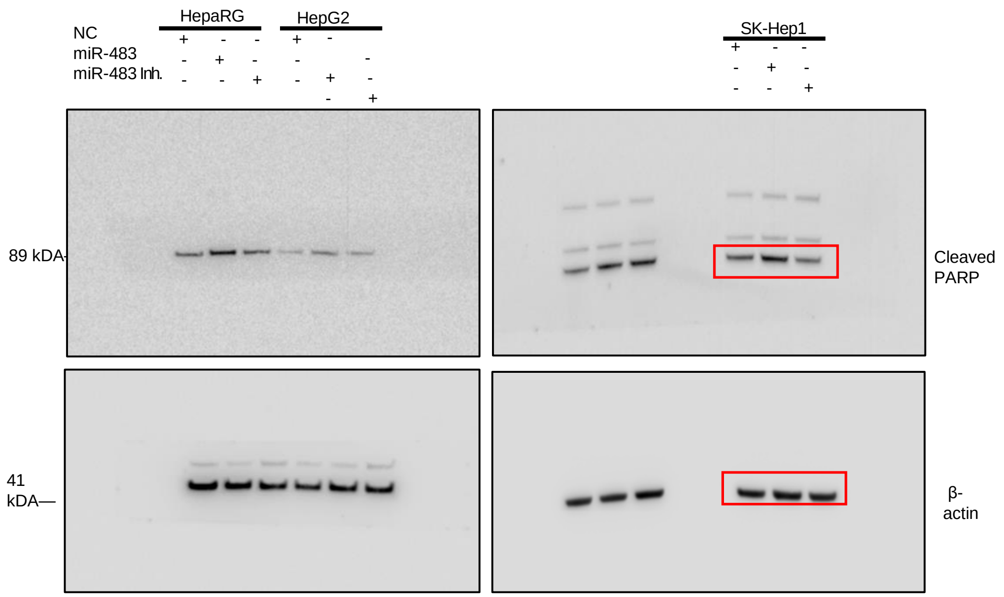

HepaRG
HepG2
SK-Hep1
NC
miR-483
miR-483 Inh.
89 kDA
41 kDA
Cleaved PARP
β-actin

Figure 4 **E**

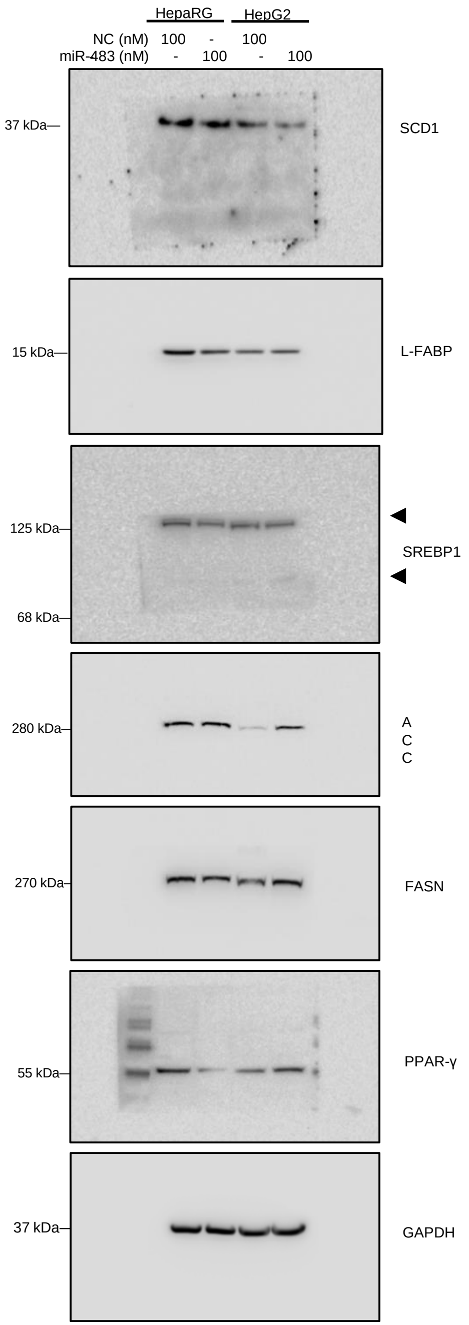
HepaRG
HepG2
NC (nM) 100 - 100
miR-483 (nM) - 100 - 100
37 kDa
SCD1
15 kDa
L-FABP
125 kDa
68 kDa
SREBP1
280 kDa
A
C
C
270 kDa
FASN
55 kDa
PPAR-γ
37 kDa
GAPDH

Figure 4

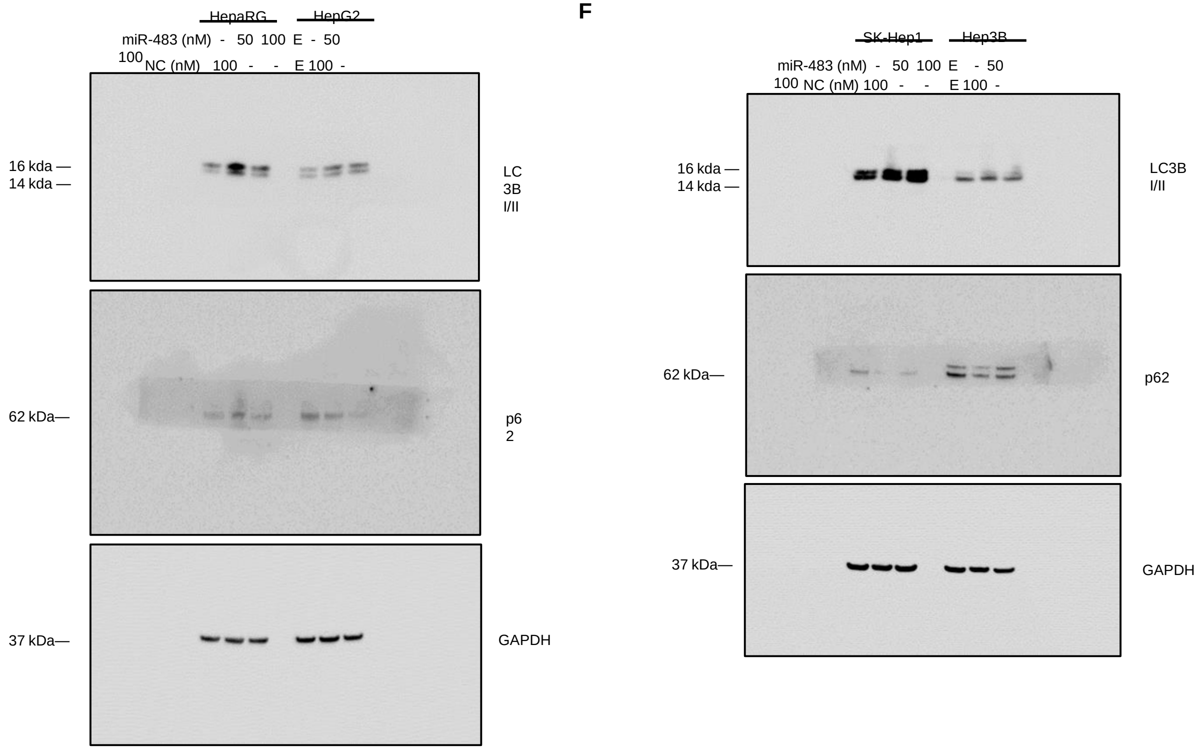
HepaRG
HepG2
miR-483 (nM) - 50 100 E - 50
100
NC (nM) 100 - - E 100 -
16 kda —
14 kda —
LC 3B I/II
62 kDa—
p6 2
37 kDa—
GAPDH
F
SK-Hep1
Hep3B
miR-483 (nM) - 50 100 E - 50
100 NC (nM) 100 - - E 100 -
16 kda —
14 kda —
LC3B I/II
62 kDa—
p62
37 kDa—
GAPDH

Figure 5 B

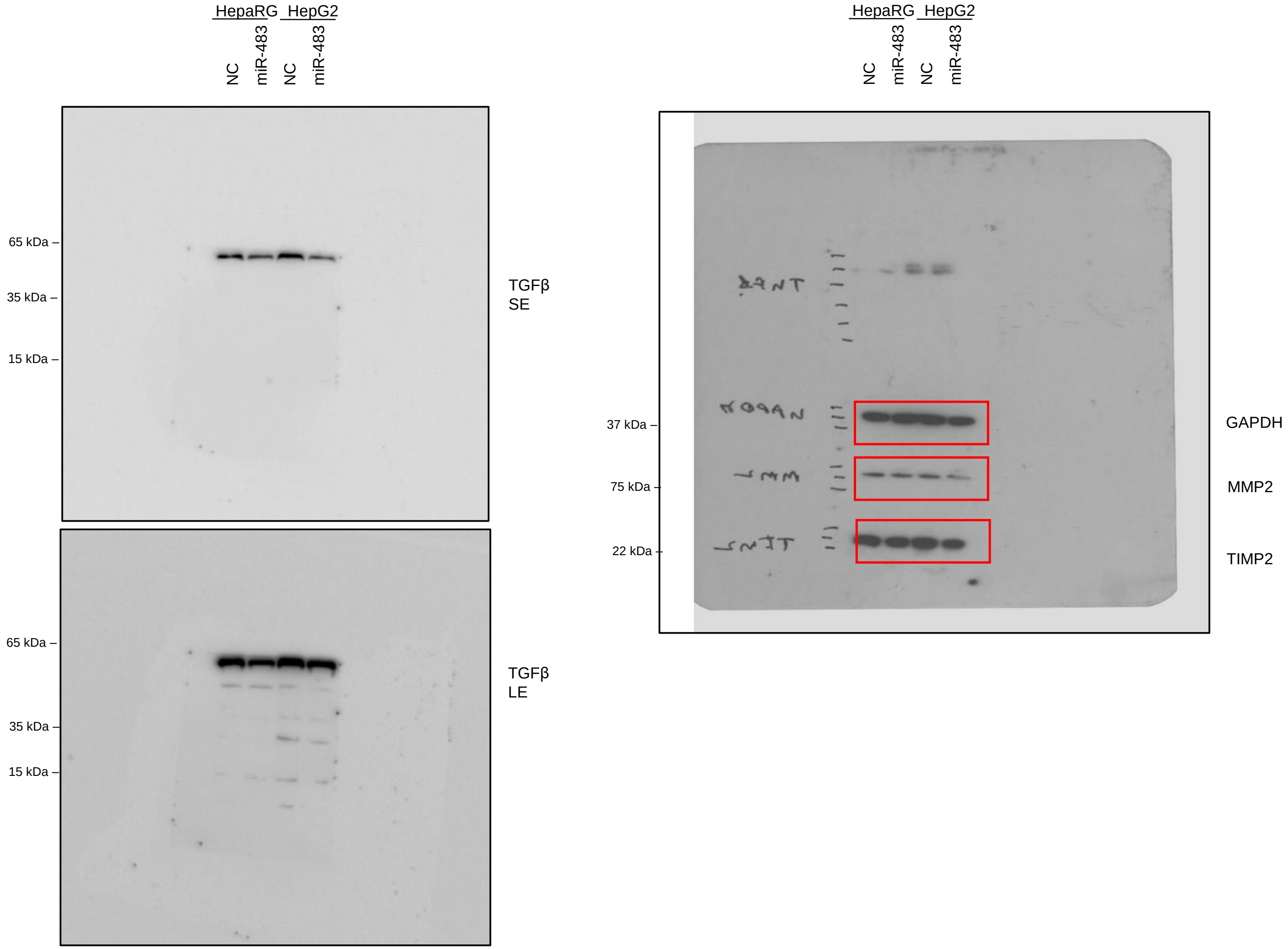

HepaRG
HepG2
NC
miR-483
NC
miR-483
65 kDa
35 kDa
15 kDa
TGFβ SE
65 kDa
35 kDa
15 kDa
TGFβ LE
HepaRG
HepG2
NC
miR-483
NC
miR-483
37 kDa
75 kDa
22 kDa
GAPDH
MMP2
TIMP2

Figure 5 D

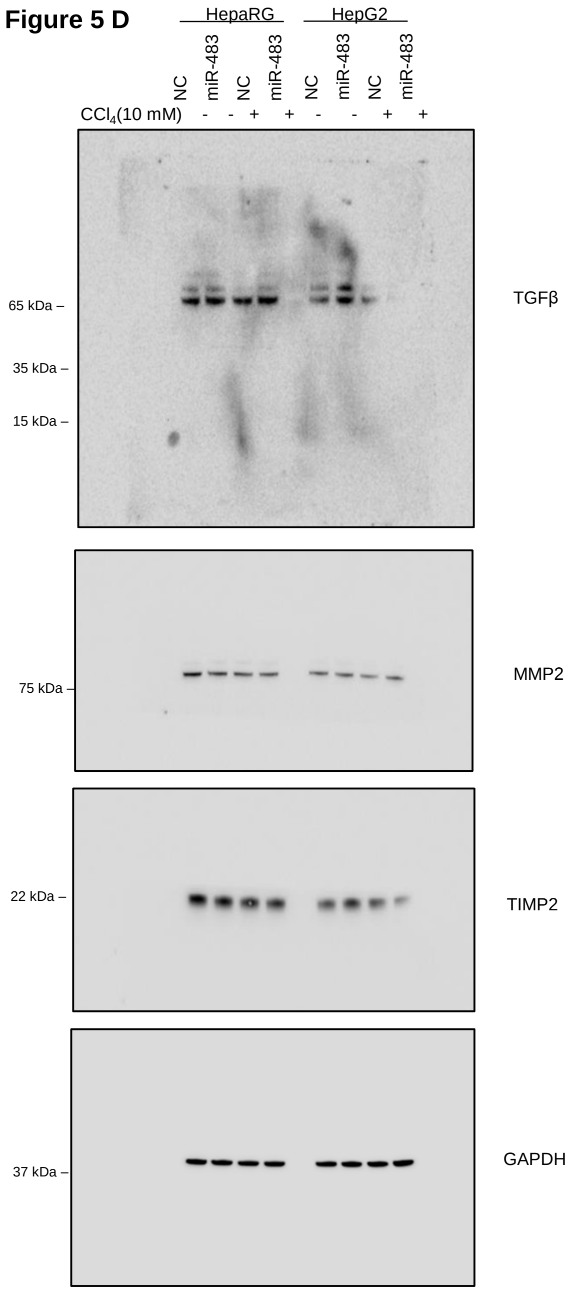

HepaRG
HepG2
NC
miR-483
NC
miR-483
NC
miR-483
NC
miR-483
CCl4(10 mM)
- - + + - - + +
65 kDa –
35 kDa –
15 kDa –
TGFβ
75 kDa –
MMP2
22 kDa –
TIMP2
37 kDa –
GAPDH

Figure 6 C

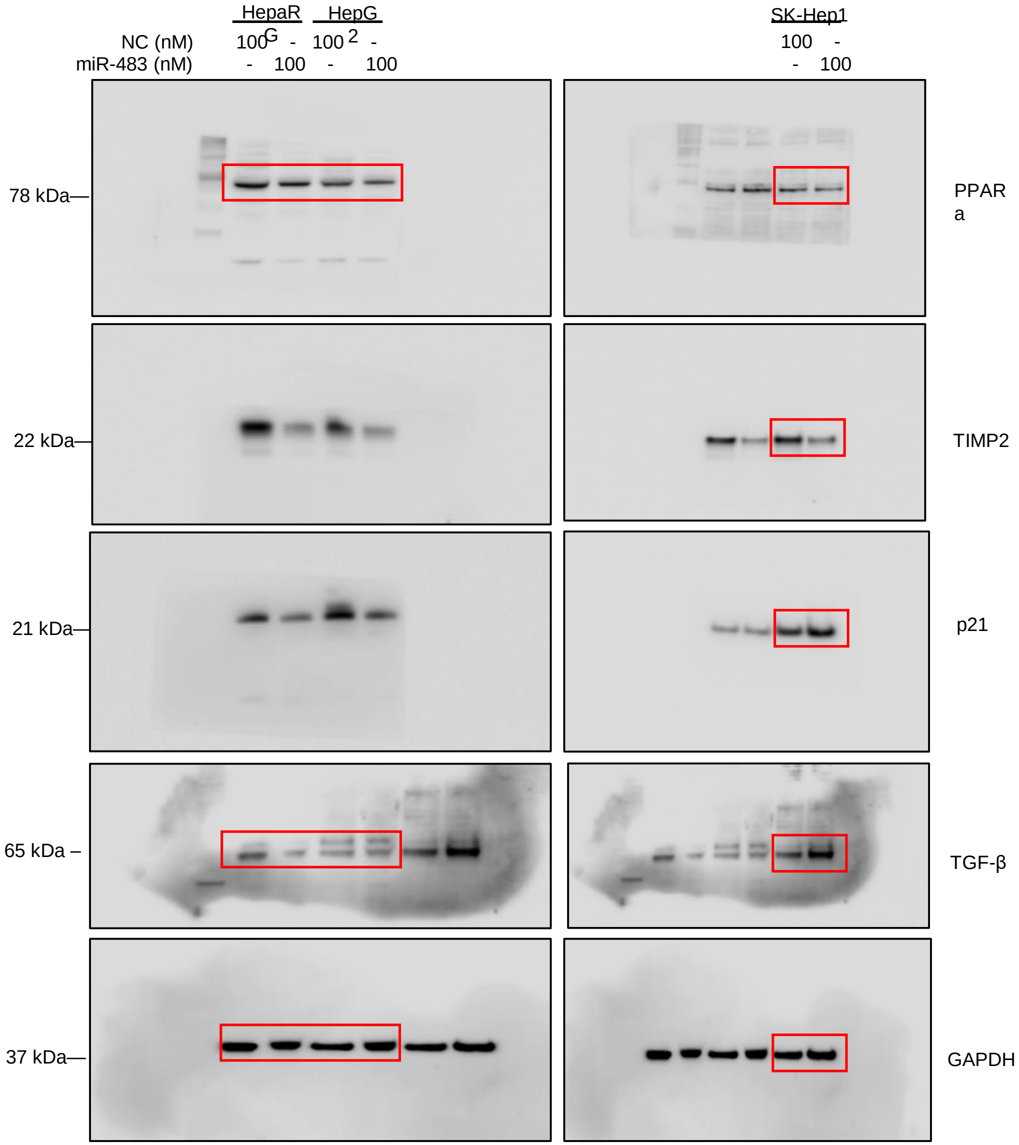
HepaRG
HepG2
SK-Hep1
NC (nM) 100 - 100 - 100 -
miR-483 (nM) - 100 - 100 - 100
78 kDa—
22 kDa—
21 kDa—
65 kDa –
37 kDa—
PPARa
TIMP2
p21
TGF-β
GAPDH
